# Estimating meaningful thresholds for multi-item questionnaires using item response theory

**DOI:** 10.1007/s11136-023-03355-8

**Published:** 2023-02-13

**Authors:** Berend Terluin, Jaimy E. Koopman, Lisa Hoogendam, Pip Griffiths, Caroline B. Terwee, Jakob B. Bjorner

**Affiliations:** 1 Department of General Practice, Amsterdam UMC Location Vrije Universiteit Amsterdam, Amsterdam, The Netherlands; 2 Amsterdam Public Health Research Institute, Amsterdam, The Netherlands; 3 Department of Plastic, Reconstructive, and Hand Surgery, Erasmus MC University Medical Center, Rotterdam, The Netherlands; 4 Hand and Wrist Center, Xpert Clinics, Eindhoven, The Netherlands; 5 Patient Centered Endpoints, IQVIA, Reading, UK; 6 Department of Epidemiology and Data Science, Amsterdam UMC Location Vrije Universiteit Amsterdam, Amsterdam, The Netherlands; 7 QualityMetric, Johnston, RI USA; 8 Department of Public Health, University of Copenhagen, Copenhagen, Denmark

**Keywords:** Meaningful threshold, Cutoff point, Item response theory (IRT), Adjusted predictive modeling, Receiver operating characteristic (ROC), Patient acceptable symptom state (PASS)

## Abstract

**Purpose:**

Meaningful thresholds are needed to interpret patient-reported outcome measure (PROM) results. This paper introduces a new method, based on item response theory (IRT), to estimate such thresholds. The performance of the method is examined in simulated datasets and two real datasets, and compared with other methods.

**Methods:**

The IRT method involves fitting an IRT model to the PROM items and an anchor item indicating the criterion state of interest. The difficulty parameter of the anchor item represents the meaningful threshold on the latent trait. The latent threshold is then linked to the corresponding expected PROM score. We simulated 4500 item response datasets to a 10-item PROM, and an anchor item. The datasets varied with respect to the mean and standard deviation of the latent trait, and the reliability of the anchor item. The real datasets consisted of a depression scale with a clinical depression diagnosis as anchor variable and a pain scale with a patient acceptable symptom state (PASS) question as anchor variable.

**Results:**

The new IRT method recovered the true thresholds accurately across the simulated datasets. The other methods, except one, produced biased threshold estimates if the state prevalence was smaller or greater than 0.5. The adjusted predictive modeling method matched the new IRT method (also in the real datasets) but showed some residual bias if the prevalence was smaller than 0.3 or greater than 0.7.

**Conclusions:**

The new IRT method perfectly recovers meaningful (interpretational) thresholds for multi-item questionnaires, provided that the data satisfy the assumptions for IRT analysis.

**Supplementary Information:**

The online version contains supplementary material available at 10.1007/s11136-023-03355-8.

## Introduction

The use of patient-reported outcome measures (PROMs) has become standard practice in clinical research and daily clinics due to the growing emphasis on patient-centered and value-based care. PROMs typically consist of multi-item questionnaires used to measure constructs (or “traits”), such as “depression” or “pain.” However, because PROM scores are often continuous scores without intrinsic meaning, there is a need for (clinically) meaningful thresholds or cutoff points to facilitate interpretation. Examples of meaningful thresholds include a diagnostic cutoff point for depression, and a patient acceptable symptom state (PASS) threshold for pain. Determining a meaningful threshold on a questionnaire requires the comparison with an external criterion indicating the presence or absence of a meaningful trait level to define an interpretable “state” (e.g., clinical depression, or an acceptable symptom state). For clarity, we provide some terminology in Box 1.

**Box 1:** Terminology

**Trait**: The construct of interest (e.g., depression or pain) that is intended to be measured by a PROM, usually a multi-item questionnaire. The construct itself is not directly observable, hence “latent.” The latent trait is usually continuous. The PROM score provides an approximation of the true trait level. PROM scores are observed (i.e., manifest) **Perceived trait**: The level of the latent trait as being perceived by the patient or by an observer (e.g., a clinician). The perceived trait is equal to the latent trait plus some random (measurement) error **State (of interest)**: A clinically meaningful condition that is characterized by a minimum level of a trait of interest. Examples of meaningful states are clinical depression and acceptable symptom state **Meaningful threshold**: The minimum trait level above which a meaningful state is assumed to exist. The meaningful threshold can be thought of as a location on the latent trait (in which case the threshold is latent), or it can be thought of as a particular PROM score (in which case the threshold is manifest, and an approximation of the latent threshold). The term “cutoff point” can be used to indicate a manifest threshold of a PROM **State assessment**: The procedure used to determine whether or not a state of interest is present. The procedure is independent of the PROM of interest. Examples of state assessments are the making of a diagnosis of clinical depression by a trained professional, and the patient response to a targeted question (often called an “anchor” question) **State scores**: The results of state assessment. Typically, state scores are dichotomous: “1” for the state of interest is present, and “0” for the state is absent **State difficulty**: The level of a trait (defining a state of interest) where the probability that a state assessment results in establishing that the state of interest is present, is 50%	

Given that depression represents a continuous trait in the general population [1], the state clinical depression can be conceptualized as a level of depression above a certain threshold on this trait. Then, making a diagnosis of clinical depression can be seen as estimating a patient’s level of depression, based on their history, and to determine whether this level is above or below the threshold of clinical depression [2]. In this example, the threshold is agreed upon by the psychiatric professional community.

The PASS represents a threshold of clinical importance beyond which patients consider their level of symptoms (e.g., pain) as acceptable [3]. A PASS threshold is typically determined using an “anchor” question like “Do you consider your current level of pain acceptable, yes or no?”. The question assumes that patients compare their perceived level of pain to a personal threshold (or benchmark) of acceptability. This PASS threshold probably differs across individuals. Thus, the best group-level PASS estimate would be the mean of the individual PASS thresholds in a group of patients.

Given a continuous “test” variable (i.e., a variable holding the PROM scores) and a dichotomous “state” variable (i.e., a variable holding the state scores), the traditional method to determine a meaningful threshold or cutoff point is receiver operating characteristic (ROC) analysis. ROC analysis examines the sensitivity and specificity of all possible test scores with respect to their ability to classify subjects with respect to the meaningful state [4]. As a cutoff point, a test score can be selected based on its desired sensitivity and/or specificity, controlling the type and amount of misclassification. Often a so-called “best” or “optimal” cutoff point is chosen of which the difference between sensitivity and specificity is minimized (top-left criterion) or the sum of sensitivity and specificity is maximized (Youden criterion [5]; in large samples with normally distributed test scores, both criteria identify the same threshold [6]). An optimal ROC threshold serves to classify subjects with the least amount of misclassification.

A problem with using ROC analysis for identifying meaningful thresholds is that an optimal ROC cutoff point depends on the prevalence of the state. For any given cutoff point, an increase in the state prevalence results in an increase of the cutoff point’s sensitivity and a decrease of its specificity, whereas a decrease in the prevalence has the opposite effect [7]. An optimal ROC-based cutoff point with a balanced sensitivity and specificity in one particular situation (with a certain prevalence) will, therefore, not be the optimal cutoff point with the same sensitivity–specificity balance in another situation. In other words, an optimal ROC cutoff point is context specific [8]. As a meaningful threshold is principally independent of the state prevalence, the optimal ROC cutoff point may not identify the meaningful threshold on a continuous construct [2]. Only if the state prevalence is 50%, the optimal ROC cutoff point will correspond to the meaningful threshold [2]. In other words, whereas the optimal ROC cutoff point performs excellently in classifying cases and non-cases with minimal misclassification *in specific situations*, it is not suitable to identify the (mean) threshold on a continuous trait, as defined by clinical or patient criteria.

An alternative to ROC analysis is predictive modeling, which involves logistic regression analysis using the state variable as the outcome and the test variable as the predictor variable [9]. The optimal cutoff point is the test score that is equally likely to occur in the state-positive group as in the state-negative group (i.e., the likelihood ratio is 1). Predictive modeling identifies about the same cutoff point as ROC analysis, but with greater precision [9]. However, like the optimal ROC cutoff point, the predictive modeling cutoff point depends on the state prevalence [10]. The prevalence-related bias in the predictive modeling cutoff point depends on the reliability of the state variable, the standard deviation (SD) of the test variable, and the point-biserial correlation between the test variable and the state variable. These parameters can be used to adjust the prevalence-related bias and recover the proper threshold across a wide range of state prevalences [11].

A third method, recently introduced, is based on item response theory (IRT) [2]. This method uses the state prevalence to estimate a meaningful threshold on the latent trait scale and subsequently determines the corresponding test score threshold. However, this method assumes perfect validity and reliability of the state scores, which is arguably questionable. It is currently unknown to which extent the reliability of the state scores affects the threshold estimate.

This paper presents an improved IRT-based method to estimate meaningful thresholds, which is based on the work of Bjorner et al. [12] in estimating meaningful within-individual change thresholds using longitudinal IRT. Like Bjorner et al. [12], the new method uses the IRT difficulty parameter of the state scores, instead of the state prevalence to find the latent trait threshold of interest. We will demonstrate the performance of this method using simulation studies and two real datasets. We will compare the results with the ROC method, the predictive modeling method [9], the adjusted predictive modeling method [11], and the “old” state prevalence IRT method [2].

## Method

### Item response theory

IRT aims to explain observed item scores by invoking an unobservable variable underlying these item scores [13]. For instance, the responses to the items of a depression scale can be thought of as being driven by an unobservable continuous variable (i.e., a latent trait) called “depression”. A popular IRT model is the graded response model (GRM) [14] that defines the probability of scoring in category *c* or above the following way:

$$\ln \left( {\frac{{P\left( {X_{ij} \ge c | \theta_{i} } \right)}}{{P\left( {X_{ij} < c | \theta_{i} } \right)}}} \right) = a_{j} \left( {\theta_{i} - b_{jc} } \right)$$

where, ${X}_{ij}$ is the response of person *i* to item *j, *${\theta }_{i}$ is the score of person *i* on the latent trait. In principle, $\theta$ can take values from $-\infty$ to + $\infty$. $\ln \left( {\frac{{P\left( {X_{ij} \ge c | \theta_{i} } \right)}}{{P\left( {X_{ij} < c | \theta_{i} } \right)}}} \right)$ is the natural logarithm of the odds of person *i* scoring *c* or higher on item *j*. ${a}_{j}$ is the discrimination parameter for item *j*. The discrimination parameter refers to the slope of the option characteristic curves, and is a measure of how well the item (categories) distinguishes respondents high and low on the trait. ${b}_{jc}$ is the difficulty parameter for category *c* on item *j*. The difficulty parameter represents the trait level where the probability of endorsing response category *c* or higher is 50%. The difficulty parameter also indicates the level of the trait where the item response option is most informative.

**Fig. 1:**
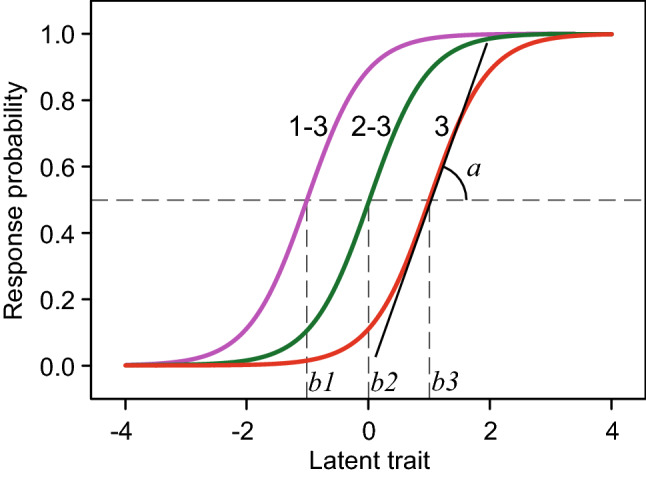
Option characteristic curves of an item with 4 ordered response options (0, 1, 2 and 3) based on the graded response model. Three curves are displayed showing, from left to right, the probability of endorsing options 1, 2 or 3 instead of option 0 (labeled “1–3”), the probability of endorsing options 2 or 3 instead of options 0 or 1 (labeled “2–3”), and the probability of endorsing option 3 instead of options 0, 1 or 2 (labeled “3”), respectively, as a function of the latent trait. The difficulty parameters (labeled “*b1*”, “*b2*” and “*b3*”) are indicated by vertical dashed lines. The discrimination parameter (labeled “*a*”) reflects the slope of the option characteristic curves

For an item with 4 response options (i.e., 0, 1, 2 and 3), Fig. 1 shows the item–trait relationship graphically as modeled using the GRM [14]. As a fitted IRT model mathematically describes the relationship between responses to the items of a scale and the $\theta$ values of the underlying trait, the model is not only able to estimate the trait level ($\theta$) for a given set of responses to the items of a questionnaire, but it is also able to estimate the expected (i.e., mean) questionnaire score (i.e., the sum or test score) for a given trait level.

**Fig. 2:**
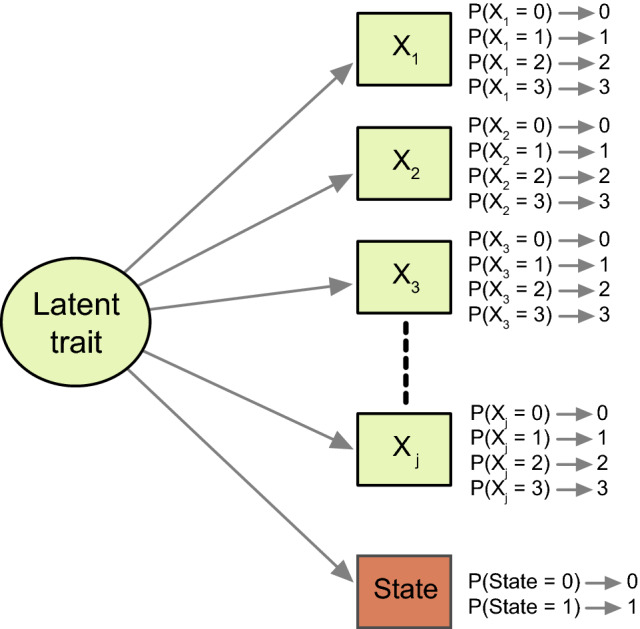
IRT model to estimate a meaningful threshold on a questionnaire with j items. Rectangles represent observed variables: questionnaire items 1 through j, (*X*_1_–*X*_j_), and the state scores (State). The oval represents the latent trait underlying the item scores (and the state scores). The latent trait determines the probabilities of scoring the item response options 0–3 (e.g., *P*(*X*_1_ = 0), etc.) and the state scores item, according to the item parameters difficulty and discrimination (not shown)

A meaningful threshold can be thought of as a threshold located somewhere on the latent trait. Such a threshold can be estimated by including the dichotomous state variable in the IRT model, effectively treating the state variable as an extra item (Fig. 2). The model for such a dichotomous item is:

$$\ln \left( {\frac{{P\left( {X_{is} = 1 | \theta_{i} } \right)}}{{P\left( {X_{is} = 0 | \theta_{i} } \right)}}} \right) = a_{s} \left( {\theta_{i} - b_{s} } \right)$$

where, $\ln \left( {\frac{{P\left( {X_{is} = 1 | \theta_{i} } \right)}}{{P\left( {X_{is} = 0 | \theta_{i} } \right)}}} \right)$ is the natural logarithm of the odds that person *i* is assessed to be in the state of interest *s*, ${a}_{s}$ is the discrimination parameter of the state variable *s*, ${b}_{s}$ is the difficulty parameter of the state variable *s*.

The logic behind this approach is that, like the questionnaire items, the state variable is an indicator of the latent trait. Adding the state variable to the IRT model yields a single option characteristic curve for the dichotomous state variable. Importantly, the model estimates a single difficulty parameter for the state variable, which represents the trait level where the probability of scoring 1 on the state variable is 50%. Interestingly, this point also represents the mean of the individual thresholds for endorsing the state item [12].^1^ Once the meaningful threshold is identified in terms of the latent trait level, the fitted IRT model provides the corresponding threshold in terms of the PROM score using the expected test score function.

### Simulations

We simulated datasets with known individual meaningful thresholds to demonstrate how the new IRT method performs, relative to the ROC method, the predictive modeling method [9], the adjusted predictive modeling method [11], and the old IRT method based on the state prevalence [2]. The beauty of simulations is that the true meaningful threshold can be specified and simulated, and the results can be judged with respect to the extent to which the truth can be accurately recovered.

**Fig. 3:**
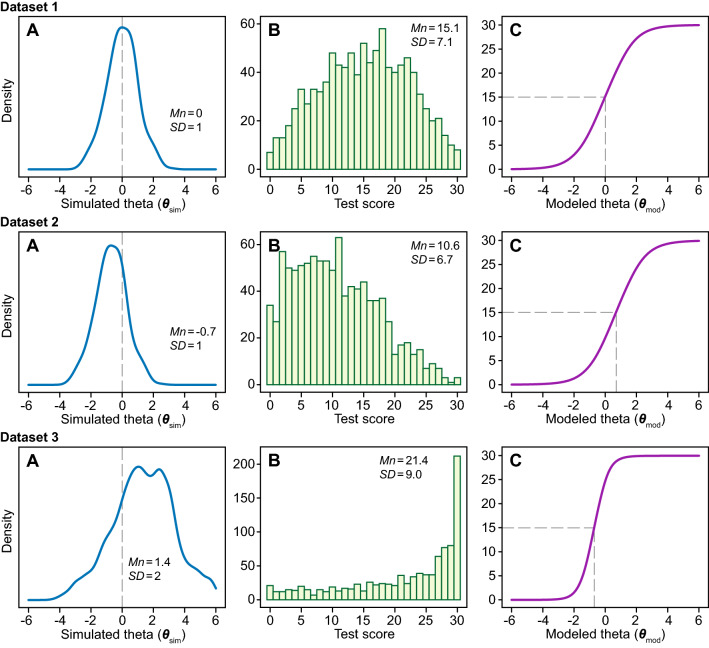
Examples of 3 simulated datasets. A-panels: Density curves showing the simulated theta distributions (*Mn* = mean, *SD* = standard deviation). B-panels: Histograms showing the distribution of the corresponding test scores (i.e., scale scores; *Mn* = mean, *SD* standard deviation). C-panels: Expected test function curves showing the expected scale score as a function of the modeled theta. Meaningful thresholds defined by ${\theta }_{sim}$ = 0 are indicated by vertical dashed lines (A-panels). The expected test scores corresponding to the meaningful thresholds are indicated by horizontal dashed lines (C-panels)

We simulated multiple datasets with 1000 subjects. We used GRM IRT to simulate item responses to a hypothetical 10-item questionnaire, each item having 4 response options, based on a prespecified set of item parameters (see Supplementary file 1, section 1) and varying distributions of the latent trait ($\theta$) (the simulation syntax is provided in Supplementary file 1, section 2). We varied the mean of the normally distributed latent trait (${\theta }_{\mathrm{sim}}$) across the values − 1.4, − 0.7, 0, 0.7 and 1.4 (thus simulating samples of low to high mean severity of the trait), and the standard deviation (SD) of ${\theta }_{\mathrm{sim}}$ across 1, 1.5 and 2 (thus simulating more and less heterogeneous samples). Figure 3 shows the distribution of the latent traits (A-panels) and the resulting distribution of the 10-item scale scores (B-panels) for three example datasets. If the mean ${\theta }_{\mathrm{sim}}$ matches the mean simulated *b*-parameter (Fig. 3, dataset 1), the scale score was normally distributed. In case of a mismatch between the mean ${\theta }_{\mathrm{sim}}$ and the mean *b*-parameter (Fig. 3, datasets 2 and 3), the scale score became skewed and might even demonstrate floor or ceiling effects, despite the underlying latent trait (${\theta }_{\mathrm{sim}}$) being normally distributed. Figure 3 also shows the expected test function curves obtained from a fitted GRM model (C-panels). By default, a GRM model assumes an underlying latent trait (denoted “modeled theta” or ${\theta }_{\mathrm{mod}}$) with a mean of zero and an SD of 1. Therefore, ${\theta }_{\mathrm{mod}}$ is a linear transformation of ${\theta }_{\mathrm{sim}}$ and a threshold on the simulated theta scale (${\theta }_{\mathrm{sim}}^{\mathrm{T}}$) corresponds to a threshold on the modeled theta scale (${\theta }_{\mathrm{mod}}^{\mathrm{T}}$) according to the following equation:

$$\theta_{\bmod }^{{T}{}} = (\theta_{sim}^{{T}{}} - mean\;(\theta_{sim} ))/SD(\theta_{sim} )$$

Note, however, that the expected test score corresponding to ${\theta }_{\mathrm{sim}}^{\mathrm{T}}$ = 0 was independent of the distribution of ${\theta }_{\mathrm{sim}}$. For illustration, we consider dataset 3 in Fig. 3. After IRT modeling and fitting the dataset, the threshold ${\theta }_{\mathrm{mod}}^{\mathrm{T}}$, following the equation above, was (0–1.4)/2 = – 0.7. Panel C shows the expected test score function of the fitted model (i.e., the relationship between ${\theta }_{\mathrm{mod}}$ and the test score). Based on the expected test score function, the threshold ${\theta }_{\mathrm{mod}}^{\mathrm{T}}$ corresponded to an expected test score of 15.1.

**Fig. 4:**
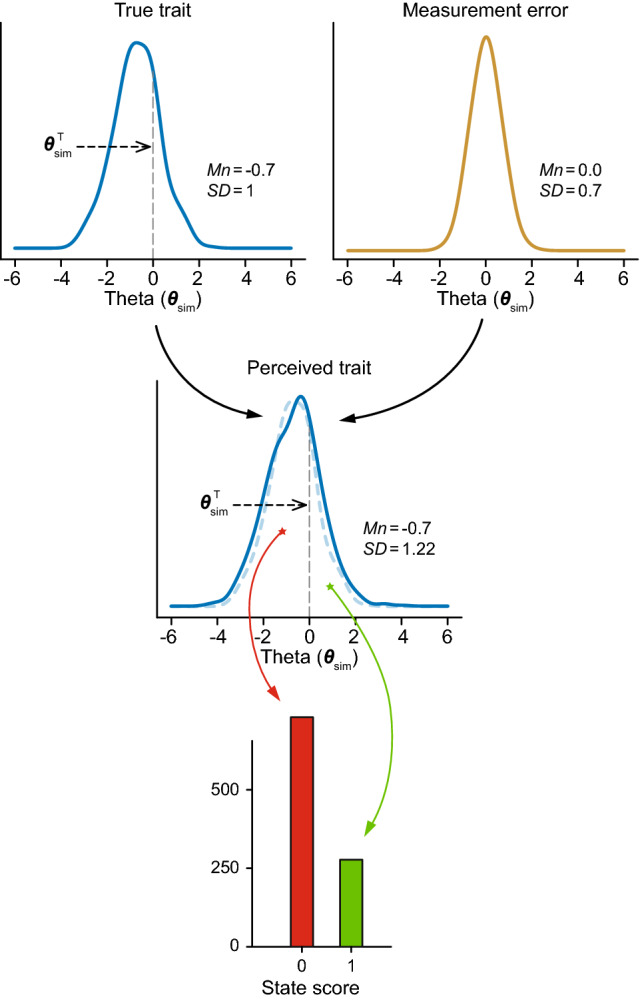
Graphical representation of how the state scores were simulated. The perceived trait is the true trait plus measurement error (all in the theta metric). In the perceived trait graph, the true trait is indicated by a dashed curve. The state scores (“1”: state of interest is present; “0”: state of interest is absent) are a discretization of the perceived trait relative to the meaningful threshold (${\theta }_{\mathrm{sim}}^{\mathrm{T}}$)

The state scores were simulated as follows. We assumed that the state assessment was based on the comparison of a “perceived trait” with the relevant threshold. Professionals making a depression diagnosis compare the perceived level of depression with the professionally defined threshold of clinical depression. Patients answering a PASS anchor question about pain compare their perceived level of pain with their personal thresholds of acceptability. The perceived trait was assumed to consist of the true trait (i.e., the latent trait ${\theta }_{\mathrm{sim}}$) and some “measurement error” (Fig. 4) [15]. The measurement error was simulated to have a normal distribution with a variance chosen in such a way as to obtain reliability values of the perceived trait of 0.5, 0.7, or 0.9. The meaningful threshold (${\theta }_{\mathrm{sim}}^{\mathrm{T}}$) was arbitrarily set to be zero for all datasets. We did not simulate variability of the thresholds across subjects, as this would only add (a little) extra error to the perceived trait. The “observed” dichotomous state scores were then obtained by comparing the continuous perceived trait with the threshold (${\theta }_{\mathrm{sim}}^{\mathrm{T}}$). Thus, the state scores were a discretization of the underlying perceived trait variable. The observed state prevalence was the proportion of subjects who’s perceived trait exceeded the threshold.

The exact true (i.e., as simulated) meaningful threshold in terms of the expected scale score, corresponding to ${\theta }_{\mathrm{sim}}^{\mathrm{T}}$ = 0, based on the simulated item parameters (see Supplementary file 1, section 1) was 15.139 (see Supplementary file 1, section 3 for details of the calculation).

### Real dataset: diagnostic thresholds

The first real dataset consists of data from a trial involving primary care patients with emotional distress or minor mental disorders [16]. At baseline, 307 patients completed the Hospital Anxiety Depression Scale (HADS), a self-report questionnaire measuring anxiety and depression [17]. In addition, standardized psychiatric diagnoses were obtained by trained interviewers using the Composite International Diagnostic Interview (CIDI) [18]. The original study was approved by the ethical committee of The Netherlands Institute of Mental Health and Addiction and all patients provided written informed consent. We used the HADS depression scale and the CIDI mild, moderate, and severe major depressive disorder (MDD) diagnoses (criteria according to the Diagnostic and Statistical Manual, Fourth Edition; DSM-IV [19]). The HADS depression scale consists of 7 items with 4 response options. Hence, the HADS depression total score ranges from 0 to 21 (0 = no depression, 21 = severe depression). We aimed to establish the clinical thresholds for mild, moderate, and severe MDD. To that end, we constructed 3 dichotomous state variables to be used in separate analyses in conjunction with the HADS items. The first state variable was used to establish the threshold for mild MDD, contrasting mild, moderate, and severe MDD (coded “1”) to no MDD (coded “0”). The second state variable was used to establish the threshold for moderate MDD, contrasting moderate and severe MDD (coded “1”) to no and mild MDD (coded “0”). The third state variable was used to establish the threshold for severe MDD, contrasting severe MDD (coded “1”) to no, mild, and moderate MDD (coded “0”).^2^

### Real dataset: patient acceptable symptom state (PASS)

The second real dataset was obtained from the Hand-Wrist Study Group cohort and comprised 3522 patients who underwent surgical trigger digit release [20, 21]. All patients were invited to complete the Michigan Hand outcomes Questionnaire (MHQ), a PROM covering six subdomains of hand function [22], three months postoperatively. The study was approved by the local medical ethical review board, and all patients provided written informed consent. We used the MHQ pain subscale, which has a score ranging from 0 to 100 (0 = worst possible pain, 100 = no pain). This score is derived from 5 items, each having five response options. To determine the PASS of the MHQ pain score, we asked patients to answer the following anchor question [23]: “How satisfied are you with your treatment results thus far?” with response options: “excellent,” “good,” “fair,” “moderate,” or “poor.” Considering that the PASS represents the threshold above which a patient is satisfied with his or her current state [3], we adopted the threshold between “fair” and “good” as the PASS and dichotomized the ratings accordingly.

### Analysis

#### Simulated samples

We calculated thresholds using the ROC method (Youden criterion) [4, 5], the predictive modeling method [9], the adjusted predictive modeling method [11], the old state prevalence IRT method [2], and the new state difficulty IRT method. Bias was calculated as the mean residual (true threshold minus estimated threshold), and the mean square residual (MSR) as the mean of the squared residuals.

#### Real datasets

As unidimensionality is an important prerequisite for IRT, we checked unidimensionality of the datasets through confirmatory factor analysis. The items were treated as categorical. The following scaled fit indices were taken as indicative of unidimensionality: comparative fit index (CFI) > 0.95, Tucker–Lewis index (TLI) > 0.95, root mean square error of approximation (RMSEA) < 0.06, and standardized root mean square residual (SRMR) < 0.08 [24]. As in the simulated samples, we calculated thresholds using the ROC method [4, 5], the predictive modeling method [9], the adjusted predictive modeling method [11], the old state prevalence IRT method [2], and the new state difficulty IRT method. 95% Confidence intervals were obtained through empirical bootstrap (1000 samples) [25].

### Software

We used the statistical program R, version 4.0.3 [26], to organize the data, calculate the predictive and adjusted thresholds, and perform bootstrapping. The pROC package, version 1.17.0.1 [27], was used to perform ROC analyses. The lavaan package, version 0.6–8 [28], was used to perform confirmatory factor analysis. The mirt package, version 1.33.2 [29], was used to simulate datasets, fit GRMs, and calculate expected test scores.

## Results

### Simulations

**Fig. 5:**
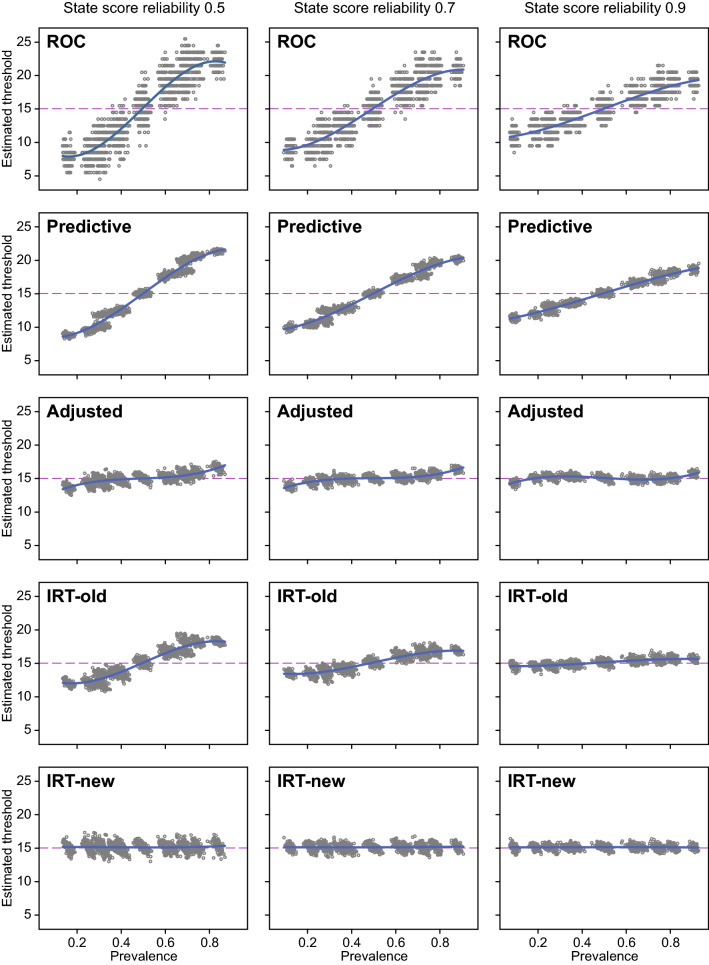
Estimated meaningful thresholds across 4500 simulated datasets by state prevalence, state scores reliability, and method (row 1: ROC, row 2: predictive modeling, row 3: adjusted predictive modeling, row 4: old IRT method using state prevalence, row 5: new IRT method using state difficulty parameter. The true threshold was 15.139 in all datasets, indicated by horizontal dashed lines

The simulated datasets varied in means and standard deviations of the test scores (Table 1). Because of the fixed meaningful threshold (${\theta }_{\mathrm{sim}}^{\mathrm{T}}$ = 0), increasing or decreasing the mean ${\theta }_{\mathrm{sim}}$ intentionally lead to increase or decrease of the state prevalence (i.e., the proportion of subjects exceeding the threshold). Moreover, as increasing or decreasing the mean ${\theta }_{\mathrm{sim}}$ caused mismatch between the mean ${\theta }_{\mathrm{sim}}$ and the mean item difficulty parameter, this inevitably caused variable degrees of skewness (as illustrated in Fig. 3). Figure 5 shows the estimated meaningful thresholds as a function of the state prevalence, by method and state scores reliability. The ROC-based thresholds and the predictive modeling-based thresholds clearly varied with the state prevalence and the state scores reliability. The old state prevalence IRT method [2] also varied with the state prevalence and the state scores reliability, although to a lesser degree. The adjusted predictive modeling method performed significantly better, although some bias remained if the state prevalence was smaller than 0.3 or greater than 0.7. In contrast to the other methods, the new state difficulty IRT method perfectly recovered the true meaningful threshold with almost no bias and high precision (Table 2). Across all simulated samples, the ROC method yielded the most prevalence-related bias and the least precision, whereas the new IRT method yielded the least bias and the greatest precision.

**Table 1:** Sample characteristics of the 4500 simulated datasets (mean, range)

Sample characteristic	Mean	Range
Mean test score	15.0	6.2; 23.9
SD test score	8.0	5.2; 10.5
Skewness test score	− 0.01	− 1.19; 1.21
Kurtosis test score	− 0.54	− 1.48; 1.25
Floor effects	0.06	0.00; 0.25
Ceiling effects	0.06	0.00; 0.26
State prevalence^a^	0.50	0.07; 0.93

^a^State prevalence based on the proportion of persons passing the threshold on the perceived trait

**Table 2:** Bias and mean square residual (MSR) by method, state scores reliability, and state prevalence

Method	Prevalence < 0.3		0.3 ≤ Prevalence < 0.5		0.5 ≤ Prevalence < 0.7		Prevalence ≥ 0.7	
Reliability 0.5	Bias	MSR	Bias	MSR	Bias	MSR	Bias	MSR
ROC	− 6.69	47.65	− 3.02	16.63	2.94	16.30	6.59	45.84
Predictive modeling	− 5.60	32.23	− 2.41	8.57	2.15	7.54	5.47	30.76
Adjusted predictive modeling	− 0.96	1.42	− 0.23	0.34	0.04	0.24	0.95	1.37
Old IRT (state prevalence)	− 2.89	8.86	− 1.35	3.26	1.28	3.10	2.95	9.09
New IRT (state difficulty)	0.10	0.54	0.02	0.40	− 0.06	0.34	0.05	0.49

Reliability 0.7	Bias	MSR	Bias	MSR	Bias	MSR	Bias	MSR
ROC	− 5.10	28.43	− 1.84	7.31	1.82	7.26	4.77	25.45
Predictive modeling	− 4.20	18.65	− 1.45	3.60	1.31	3.13	4.02	17.13
Adjusted predictive modeling	− 0.65	0.74	− 0.08	0.17	− 0.04	0.14	0.56	0.67
Old IRT (state prevalence)	− 1.52	2.64	− 0.54	0.82	0.56	0.79	1.53	2.63
New IRT (state difficulty)	0.02	0.23	0.04	0.23	− 0.01	0.19	0.03	0.25

Reliability 0.9	Bias	MSR	Bias	MSR	Bias	MSR	Bias	MSR
ROC	− 3.39	13.09	− 1.10	2.91	0.92	2.59	3.26	12.21
Predictive modeling	− 2.88	8.87	− 0.92	1.47	0.79	1.24	2.77	8.22
Adjusted predictive modeling	− 0.12	0.25	0.13	0.10	− 0.21	0.13	0.03	0.21
Old IRT (state prevalence)	− 0.49	0.37	− 0.13	0.16	0.15	0.16	0.50	0.38
New IRT (state difficulty)	− 0.01	0.13	0.03	0.10	− 0.03	0.10	0.00	0.13

### Real dataset: diagnostic thresholds

The sample characteristics are shown in Table 3. The prevalence of any MDD (i.e., mild, moderate, and severe MDD) was 49%. The mean HADS depression score was 10.7. The fit indices showed some violation of the unidimensionality assumption; however, none of the (absolute) residual correlations exceeded 0.2. The reliability of the diagnostic variable, expressed as the variance of the diagnosis explained by the latent depression trait as measured by the HADS [30], was 0.34. The estimated thresholds for mild, moderate, and severe MDD, using different methods, are shown in Table 4. As the prevalence of any MDD was close to 50%, the threshold for mild MDD should be close to the mean score in the sample [10]. This was confirmed for most methods; only the estimated ROC threshold was lower than the mean sample score. For the other thresholds, with state prevalences < 50%, the methods diverged as expected. The new IRT method identified 10.6, 15.4, and 18.2 as the thresholds for mild, moderate, and severe MDD. The adjusted predictive modeling method identified practically the same thresholds for mild and moderate MDD, but, compared to the new IRT method, the adjusted method slightly underestimated the threshold for severe MDD while its precision was slightly less than the new IRT method.

**Table 3:** Sample and scale characteristics of the HADS dataset (*N* = 295)

Characteristics	Values
Gender (proportion females)	0.60
Age, mean (SD)	39.5 (9.2)
Prevalence mild MDD^b^	0.23
Prevalence moderate MDD^b^	0.12
Prevalence severe MDD^b^	0.14
HADS^a^ depression score, mean (SD)	10.7 (4.3)
Scaled comparative fit index (CFI)	0.981
Scaled Tucker-Lewis index (TLI)	0.971
Scaled root mean square error of approximation (RMSEA)	0.089
Standardized root mean square residual (SRMR)	0.044
State reliability of the diagnostic variable	0.34

^a^*HADS*  Hospital Anxiety Depression Scale

^b^*MDD*  major depressive disorder (DSM-IV)

**Table 4:** Thresholds for mild, moderate, and severe MDD for the HADS depression scale

Method	Mild MDD^a^		Moderate MDD^a^		Severe MDD^a^	
	Estimate	95% CI	Estimate	95% CI	Estimate	95% CI
ROC	9.5	9.5; 10.5	10.5	9.5; 13.5	11.5	10.5; 13.5
Predictive modeling	10.8	10.3; 11.2	11.6	11.1; 12.2	12.2	11.7; 12.8
Adjusted predictive modeling	10.8	10.0; 11.7	15.3	14.1; 16.9	17.6	15.8; 20.2
Old state prevalence IRT method	10.7	10.1; 11.3	13.3	12.6; 14.1	15.2	14.3; 16.1
New state difficulty IRT method	10.6	9.7; 11.6	15.4	14.2; 16.8	18.2	16.8; 19.5

^a^*MDD* major depressive disorder (DSM-IV)

*HADS*  Hospital Anxiety Depression Scale

### Real dataset: patient acceptable symptom state (PASS)

Complete data at three months postoperatively were available for 2634 patients. The sample characteristics are depicted in Table 5. Sixty-three percent of patients were satisfied with the treatment result. The mean MHQ pain score was 71 with an SD of 23. The distribution of the pain scores was skewed to the left (skewness -0.45, ceiling effect 0.17). Confirmatory factor analysis indicated an RMSEA of 0.109, while the other fit indices and the residual correlations indicated unidimensionality. Therefore, we assumed essential unidimensionality of the scale. The estimated thresholds for the PASS, using different methods, are shown in Table 6. As expected, the state prevalence greater than 50% resulted in divergent PASS thresholds for the different methods. The new IRT method identified a PASS threshold for MHQ pain of 59.6 (95% CI 57.3; 61.7). Despite the non-normality of the MHQ pain scores, the threshold identified by the adjusted predictive modeling approach was not significantly different and of similar precision. Based on these results, it is safe to assume that the PASS threshold for the MHQ pain score (as anchored by good/excellent satisfaction with treatment results) three months after trigger finger release is around 60 (58–62). All other methods overestimated the PASS threshold due to prevalence-related bias.

**Table 5:** Sample and scale parameters of the Hand-Wrist Study Group dataset (*N* = 2634)

Characteristics	Values
Gender (proportion females)	0.67
Age, mean (SD)	59 (10)
Satisfaction with treatment results (proportions)	
Poor	0.03
Moderate	0.11
Fair	0.23
Good	0.40
Excellent	0.23
MHQ^a^ pain score, mean (SD)	71 (23)
Scaled comparative fit index (CFI)	0.993
Scaled Tucker-Lewis index (TLI)	0.989
Scaled root mean square error of approximation (RMSEA)	0.109
Standardized root mean square residual (SRMR)	0.029
State reliability of the anchor question	0.40

^a^*MHQ*  Michigan Hand outcomes Questionnaire

**Table 6:** PASS thresholds for the MHQ pain scale

	Estimate	95% CI
ROC	77.5	72.5; 77.5
Predictive modeling	69.0	68.2; 69.9
Adjusted predictive modeling	60.1	58.4; 61.8
Old state prevalence IRT method	64.3	63.0; 65.8
New state difficulty IRT method	59.6	57.3; 61.7

*MHQ*  Michigan Hand outcomes Questionnaire

## Discussion

As the use of PROMs has become standard practice in clinical research and daily clinical practice, there is an increased incentive to develop meaningful thresholds to accurately interpret questionnaire scores and facilitate clinical decision making. In this article, we have introduced a new IRT approach to estimate meaningful thresholds. The method perfectly recovered the true (as simulated) meaningful threshold as a fixed value on the latent trait with practically no bias and high precision, regardless of the state prevalence or the state scores reliability. In contrast, most of the other methods examined produced biased threshold estimates if the state prevalence was ≠ 0.5.

Importantly, meaningful thresholds or cutoff points are used for two goals that are principally incompatible with each other: interpretation and classification. The first goal, the interpretation of test scores, relates to questions such as the cutoff point for clinical depression on a depression scale, or the minimum level of acceptability on a pain scale. Interpretational thresholds, especially if they are based on relatively subjective criteria, may depend on specific sample characteristics. For instance, more severe patients may be willing to accept higher levels of knee pain and dysfunction as acceptable than less severe patients [31]. If the thresholds vary, they do so on the patient level, affecting the mean threshold in the sample. The thresholds do not vary with the prevalence of the state of interest. Our new state difficulty IRT method identifies these interpretational thresholds.

The second goal is classification of individual patients. For instance, for screening we often want thresholds that ensure the best balance between sensitivity and specificity, in order to minimize misclassification. To that end, classificational thresholds must be prevalence specific, because a cutoff point’s sensitivity and specificity change with prevalence [7]. ROC analysis identifies a test’s optimal cutoff point in a particular situation, which cannot be generalized to situations with differing prevalence and disease spectrum. Therefore, the ROC cutoff point does not identify the interpretational threshold on the latent trait (unless the prevalence is 0.5) [2].

Apart from the new state difficulty IRT method, the adjusted predictive modeling method also accurately identified the interpretational threshold with high precision, although some bias occurred with state prevalences smaller than 0.3 or greater than 0.7. This bias is at least partly due to the low or high state prevalence [11], but skewness of the test scores might also play a role. However, the observation of highly similar threshold estimates obtained by the adjusted predictive modeling method and the new IRT method, despite profound skewness and ceiling effects in our second dataset, is a promising finding. Nevertheless, future (simulation) studies should determine to what extent non-normality of the test scores affects the results of the adjusted predictive modeling approach.

The new state difficulty IRT method assumes that the state of interest can be regarded as an effect indicator [32] of the latent trait and, therefore, can be included as an additional item in the IRT model. In some cases, states may alternatively be conceptualized as having a causal effect on the latent trait. Use of such causal indicators [32] is beyond the current paper but can be handled by fitting explanatory IRT models [33].

Both the new state difficulty IRT method and the adjusted predictive modeling method can be used to estimate meaningful thresholds, but the methods come with different assumptions. For the new IRT method, the data should be unidimensional enough to allow IRT analysis [34], and the questionnaire should fit an IRT model. Although any IRT model may be employed, the GRM usually provides good fit to PROM data. Furthermore, the IRT method assumes that the latent trait is normally distributed. Skewness of the observed test scores is no problem as long as the latent trait is assumably normal. On the other hand, the adjusted predictive modeling method assumes normality of the test scores [11].

Taking these assumptions into account, the choice of method may depend on the questionnaire’s dimensionality, the distribution of the test scores, and the fit of an IRT model. In case of normally distributed test scores, both the adjusted predictive modeling method and the new IRT method may be used. If the data show profound ceiling or floor effects, we recommend using the new state difficulty IRT method. The old state prevalence IRT method [2] is clearly inferior to the new IRT method because the state prevalence is affected by the (un)reliability of the state scores. Therefore, we recommend not to use the old state prevalence IRT method [2] anymore. Similarly, ROC analysis should no longer be used to identify interpretational thresholds.

## Conclusion

We have introduced a new IRT approach to identify meaningful thresholds for multi-item questionnaires through identifying the latent trait level of the threshold of interest and linking this to the corresponding meaningful threshold on the questionnaire scale. The new IRT method is superior to the adjusted predictive modeling method, especially if the prevalence is < 0.3 or > 0.7. Therefore, we recommend to use the new IRT method to estimate meaningful (interpretational) thresholds whenever possible. The adjusted predictive modeling method is a feasible alternative in certain circumstances, for example when the PROM score is not unidimensional enough to allow IRT analysis. We provide the R-code for the new IRT method in Supplementary file 1, section 4.

## Supplementary Information

Below is the link to the electronic supplementary material.

Supplementary file1 (PDF 277 KB)

Supplementary file2 (TXT 9 KB)

## Data Availability

The HADS depression data are available in Supplementary file 2.The MHQ data are available on request (to be send to Lisa Hoogendam, email: l.hoogendam@erasmusmc.nl).

## Footnotes

1 At the location of the mean of the individual thresholds (given a normal distribution of those thresholds) 50% of the individual thresholds are smaller than the $\theta$-value at that location. Hence, for a random sample of respondents who are sitting exactly at the location of the mean of the individual thresholds, the probability that they have passed their individual threshold is 50%. Therefore, the probability that they endorse the state variable at that location is 50%. In other words, the difficulty of the state variable represents the meaningful threshold of interest.

2 The state variable with 4 diagnostic categories could also be processed as a single-state variable with 4 categories in a single analysis in conjunction with the HADS items. In that case, the state variable provides 3 difficulty estimates representing the thresholds for mild, moderate, and severe MDD on the latent trait metric.
