# Supplementary material for: Estimating meaningful thresholds for multi-item questionnaires using item response theory
Source: Qual Life Res. 2023 Feb 13;32(6):1819–30. doi: 10.1007/s11136-023-03355-8 (PMC10172229; doi:10.1007/s11136-023-03355-8)
Supplement: Supplementary file 1 — Supplementary file1 (PDF 277 KB) [file 11136_2023_3355_MOESM1_ESM.pdf]

Supplementary material to:

Estimating meaningful thresholds for multi-item questionnaires using item response theory

Berend Terluin, Jaimy E Koopman, Lisa Hoogendam, Pip Griffiths, Caroline B Terwee, Jakob B Bjorner

1. Item parameters for simulation 2

2. Simulation syntax (R-code) 3

3. Calculation of the true threshold in terms of the expected test score 14

4. R-code for estimating a meaningful threshold using IRT 15

1. Item parameters for simulation

	Discrimination	Difficulty		
Item	*a*	*b1*	*b2*	*b3*
1	1.30	-1.60	-0.80	0.40
2	1.96	-2.00	-0.80	0.10
3	0.98	-1.30	-0.40	0.40
4	1.63	-1.30	-0.40	0.70
5	2.28	-1.00	0.00	1.00
6	2.28	-0.80	0.00	1.10
7	1.96	-0.70	0.40	1.60
8	1.63	-0.80	0.40	1.30
9	1.30	-0.30	0.80	1.80
10	0.98	-0.20	0.80	1.60
Mean	1.63	-1.00	0.00	1.00

2. Simulation syntax (R-code)

```
library(mirt)
library(lavaan)
library(e1071)
library(ggplot2)
library(psych)
library(pROC)

##### Create set of item parameters

N <- 1000                  # simulated sample size
nitems <- 10               # number of items
ncat <- 4                  # number of response categories per item

a  <- c(0.6,0.6,0.8,0.8,1,1,1.2,1.2,1.4,1.4)
b2 <- (a-1)*2
bc <- (a-1)/2

set.seed(12345)

a <- sample(a)
b1 <- b2 - 1 + sample(bc)
b3 <- b2 + 1 + sample(bc)

# Adjust a to get the desired reliability

a1 <- a*1.63         # Creates reliability of ~0.85

cf.simb <- data.frame(a1,b1,b2,b3)
round(cf.simb, 3)
round(colMeans(cf.simb), 3)

( mean.b <- mean(as.matrix(cf.simb[,-1])) )
( sd.b <- sd(as.matrix(cf.simb[,-1])) )

# Transform b-parameters to d-parameters
# difficulty (b) = easiness (d) / -a

cf.sim1 <- cf.simb

colnames(cf.sim1) <- c("a1","d1","d2","d3")

cf.sim1$d1 <- -cf.simb$b1*a1
cf.sim1$d2 <- -cf.simb$b2*a1
```

```
cf.sim1$d3 <- -cf.simb$b3*a1
round(cf.sim1, 3)              # dysplays the item parameters
round(colMeans(cf.sim1), 3)

# Simulate dataset using 'mirt'

a1 <- as.matrix(cf.sim1[ , 1])
d1 <- as.matrix(cf.sim1[ , -1])

# Check reliability
# Create dataset with theta = N(0,1)

Y <- rnorm(N*10, 0, 1)
Y <- (Y-mean(Y))/sd(Y)       # set mean = 0 and SD = 1
theta.org <- as.matrix( Y )

dat <- simdata(a1, d1, N*10, itemtype="graded", Theta=theta.org)
dat <- as.data.frame(dat)

alpha(dat)$total$raw_alpha

##########################################################

# Calculate the true expected test score (ETS) corresponding to theta=0
# from the item parameters

( apars <- cf.simb[,1] )     # a parameters
( bpars <- cf.simb[,2:4] )   # b parameters

p1 <- numeric(nitems)     # probability of X = 1
p2 <- numeric(nitems)     # probability of X = 2
p3 <- numeric(nitems)     # probability of X = 3
eis <- numeric(nitems)    # expected item score

th = 0                    # theta of threshold

# p1p = probability of X >= 1
# p2p = probability of X >= 2

for(j in 1:nitems)  {
( p1p <- exp(apars[j]*(th-bpars[j,1]))/(1+exp(apars[j]*(th-bpars[j,1]))) )
( p2p <- exp(apars[j]*(th-bpars[j,2]))/(1+exp(apars[j]*(th-bpars[j,2]))) )
( p3[j]  <- exp(apars[j]*(th-bpars[j,3]))/(1+exp(apars[j]*(th-bpars[j,3]))) )
( p2[j] <- p2p-p3[j] )
( p1[j] <- p1p-p2p )
( eis[j] <- p1[j]*1 + p2[j]*2 + p3[j]*3 )
```

```
}

round(cbind(p1,p2,p3,eis),3)

sum(eis)    # Expected test score


#########################################################

##### Actual simulations

## Set parameters and numbers (of subjects and samples)

par.mn.sim <- c(-1.4, -0.7, 0, 0.7, 1.4)
par.sd.sim <- c(1, 1.5, 2)
par.rel.state <- c(0.5, 0.7, 0.9)

nr=100       # set number of times each combination must be repeated

npc <- length(par.mn.sim) * length(par.sd.sim) * length(par.rel.state)
npc          # total number of combinations
ns <- npc * nr
ns           # total number of simulated samples

## Variables to hold the results

mn.sim.par    <- as.numeric(rep(NA, ns))
sd.sim.par    <- as.numeric(rep(NA, ns))
rel.state.par <- as.numeric(rep(NA, ns))
mn.sum        <- as.numeric(rep(NA, ns))
sd.sum        <- as.numeric(rep(NA, ns))
flor          <- as.numeric(rep(NA, ns))
ceil          <- as.numeric(rep(NA, ns))
skew.sum      <- as.numeric(rep(NA, ns))
kurt.sum      <- as.numeric(rep(NA, ns))
mn.sim        <- as.numeric(rep(NA, ns))
sd.sim        <- as.numeric(rep(NA, ns))
prev.tru      <- as.numeric(rep(NA, ns)) # thrue prevalence
prev.obs      <- as.numeric(rep(NA, ns)) # observed prevalence
alpha         <- as.numeric(rep(NA, ns)) # Cronbach's alpha
cor.state.sum <- as.numeric(rep(NA, ns)) # point biserial correlation
rel.state     <- as.numeric(rep(NA, ns)) # state reliability
thr.rocy      <- as.numeric(rep(NA, ns)) # threshold based on ROC (Youden)
thr.pred      <- as.numeric(rep(NA, ns)) # threshold based on predictive modeling
thr.adj       <- as.numeric(rep(NA, ns)) # threshold based on adjusted pred.
modeling
```

```
thr.thet.pls  <- as.numeric(rep(NA, ns)) # theta thr based on plausible PVs
thr.ets.pls   <- as.numeric(rep(NA, ns)) # threshold based on old IRT method
thr.thet.irt  <- as.numeric(rep(NA, ns)) # theta thr based on new IRT method
thr.ets.irt   <- as.numeric(rep(NA, ns)) # threshold based on new IRT method
cfa.check     <-  logical(ns)            # Check admissibility of CFA solution

set.seed(1234)
index <- 0

for(k1 in 1:length(par.mn.sim))    {
for(k2 in 1:length(par.sd.sim))    {
for(k3 in 1: length(par.rel.state)) {
for(k4 in 1:nr)                    {

index <- (k1-1)*length(par.sd.sim)*length(par.rel.state)*nr +
         (k2-1)*length(par.rel.state)*nr +
         (k3-1)*nr + k4

print(index)

( mn.sim.par[index]   <- par.mn.sim[k1] )
( sd.sim.par[index]   <- par.sd.sim[k2] )
( rel.state.par[index] <- par.rel.state[k3] )

Y <- rnorm(N, 0, 1)
theta.org <- as.matrix( Y )
theta.sim <- theta.org*par.sd.sim[k2] + par.mn.sim[k1]   # transform theta.sim

( mn.sim[index]   <- mean(theta.sim)  )
( sd.sim[index]   <- sd(theta.sim)    )

   dat <- simdata(a1, d1, N, itemtype="graded", Theta=theta.sim)
   dat <- as.data.frame(dat)

   sumscore <- rowSums(dat)

(   alpha[index]  <- alpha(dat)$total$raw_alpha  )
(   mn.sum[index] <- mean(sumscore)   )
(   sd.sum[index] <- sd(sumscore)     )
(   flor[index] <- table(sumscore)[1] / length(sumscore)   )
(   ceil[index] <- table(sumscore)[length(table(sumscore))] / length(sumscore) )
(   skew.sum[index] <- skewness(sumscore, type=2)  )
(   kurt.sum[index] <- kurtosis(sumscore, type=2)  )

# Perceived trait
```

```
( rel.st <- par.rel.state[k3] )     # reliability of the 'perceived trait'

sd.error <- sqrt(((1-rel.st)/rel.st)*sd(theta.sim)^2)
theta.err <- theta.sim + rnorm(N, 0, sd.error)

var(theta.sim)/var(theta.err)  # check reliability of perceived trait

mean(theta.sim)
sd(theta.sim)
mean(theta.err)
sd(theta.err)

# true prevalence
state <- numeric(N)
state[theta.sim > 0] <- 1
( prev.tru[index] <- mean(state) )

# observed prevalence
state <- numeric(N)
state[theta.err > 0] <- 1
( prev.obs[index] <- p <- mean(state) )

## Point biserial correlation
(   cor.state.sum[index] <- cor(state, sumscore)  )


### old IRT method - plausible values

cap <- capture.output( mod <- mirt(data=dat, model=1,
                              itemtype="graded", TOL=.001) )

# plausible values

n.pls <- 10

fs.mod <- fscores(mod, plausible.draws = n.pls)

theta.thr     <- numeric(n.pls)
exp.testscore <- numeric(n.pls)

for (i in 1:n.pls)   {
theta.thr[i] <- quantile(fs.mod[[i]], prob=(1-p))
Theta <- as.matrix( theta.thr[i] )
exp.testscore[i]  <- expected.test(mod, Theta)
}
```

```
( thr.thet.pls[index]  <- mean(theta.thr) )
( thr.ets.pls[index] <- mean(exp.testscore) )


# Estimate reliability of diagnosis: CFA analysis

datw <- data.frame(dat, state)

model <- '
      F1 =~ Item_1+Item_2+Item_3+Item_4+Item_5+Item_6+
            Item_7+Item_8+Item_9+Item_10+state
      '

   fit <- cfa(model, data=datw, ordered=T, std.lv=T)

   ( cfa.check[index] <- lavInspect(fit, what="post.check") )

   # Estimate state reliability
   pe <- parameterEstimates(fit, rsquare=T)
   ( rel.state[index] <- pe$est[pe$lhs=="state" & pe$op=="r2"] )    # R-squared

rm(fit)

## ROC analysis

rocobj <- roc(state, sumscore, quiet = TRUE)
mic.roc <- coords(rocobj, x="best", input="threshold", ret="threshold",
           best.method="youden", transpose = TRUE)
( thr.rocy[index] <- mic.roc[sample(length(mic.roc),1)] )

rm(rocobj)

## Predictive modeling method / adjusted predictive modeling method

mylogit <- glm(state ~ sumscore, family = "binomial")
C <- coef(mylogit)[1]                 # intercept coefficient C
B <- coef(mylogit)[2]                 # regression coefficient B
q <- log(p/(1-p))                     # q = logodds(pre)
( thr.pred[index] <- (q-C)/B )        # predictive modeling threshold

# Adjusted predictive modeling method
rf <- (0.8/rel.state[index] - 0.5) * sd.sum[index] * cor.state.sum[index]
( thr.adj[index] <- thr.pred[index] - rf * q )

rm(mylogit)
```

```
## New IRT method

cap <- capture.output( modw <- mirt(data=datw, model=1,
                              itemtype="graded", TOL=.001) )

cf <- coef(modw, simplify=TRUE, IRTpars=TRUE)$items

( thr.thet.irt[index]  <- Theta <- as.matrix( cf[nitems+1, 2] ) )
( thr.ets.irt[index]   <- expected.test(modw, Theta, which.items = 1:10) )

rm(modw)

}
}
}
}


#####

df <- data.frame(
mn.sim.par,
sd.sim.par,
rel.state.par,
mn.sum,
sd.sum,
flor,
ceil,
skew.sum,
kurt.sum,
mn.sim,
sd.sim,
prev.tru,
prev.obs,
alpha,
cor.state.sum,
rel.state,
thr.rocy,
thr.pred,
thr.adj,
thr.thet.pls,
thr.ets.pls,
thr.thet.irt,
thr.ets.irt,
cfa.check )
```

```
write.table(df, file = "E:/Simulation-results.txt", sep = " ", row.names = F,
col.names = T)

fix(df)


######################################################################

##### Analysis of simulated data

df <- read.table(file.choose(), header=T)
## Read in: Simulation-results.txt

data.frame(names(df))
dim(df)

table(df$rel.state.par)
table(df$cfa.check)


##### SCATTERPLOTS

### Create sub-files

res <- df[df$rel.state.par==0.9, ]
nrow(res)

# threshold based on ROC Youden
ggplot(res,
    aes(x=prev.obs, y=thr.rocy)) +
    geom_point(shape=1, colour="grey50") +  # Use open circles
    geom_smooth(method="lm", se=FALSE,
    formula = y ~ poly(x, 3),
    size = 1) +      # Defines regression line
    dev.new(width=5, height=4) +
    scale_x_continuous(limits=c(0.07, 0.93), breaks=c(0.2,0.4,0.6,0.8)) +
    scale_y_continuous(limits=c(4, 26), breaks=c(5,10,15,20,25))

# threshold based on predictive modeling
ggplot(res,
    aes(x=prev.obs, y=thr.pred)) +
    geom_point(shape=1, colour="grey50") +  # Use open circles
    geom_smooth(method="lm", se=FALSE,
    formula = y ~ poly(x, 3),
    size = 1) +      # Defines regression line
```

```
    dev.new(width=5, height=4) +
    scale_x_continuous(limits=c(0.07, 0.93), breaks=c(0.2,0.4,0.6,0.8)) +
    scale_y_continuous(limits=c(4, 26), breaks=c(5,10,15,20,25))

# threshold based on (improved) adjusted predictive modeling
ggplot(res,
    aes(x=prev.obs, y=thr.adj)) +
    geom_point(shape=1, colour="grey50") +  # Use open circles
    geom_smooth(method="lm", se=FALSE,
    formula = y ~ poly(x, 3),
    size = 1) +      # Defines regression line
    dev.new(width=5, height=4) +
    scale_x_continuous(limits=c(0.07, 0.93), breaks=c(0.2,0.4,0.6,0.8)) +
    scale_y_continuous(limits=c(4, 26), breaks=c(5,10,15,20,25))

# threshold based on *&*uantile of the plausible values
ggplot(res,
    aes(x=prev.obs, y=thr.ets.pls)) +
    geom_point(shape=1, colour="grey50") +  # Use open circles
    geom_smooth(method="lm", se=FALSE,
    formula = y ~ poly(x, 3),
    size = 1) +      # Defines regression line
    dev.new(width=5, height=4) +
    scale_x_continuous(limits=c(0.07, 0.93), breaks=c(0.2,0.4,0.6,0.8)) +
    scale_y_continuous(limits=c(4, 26), breaks=c(5,10,15,20,25))

# threshold based on IRT (Method Jakob)
ggplot(res,
    aes(x=prev.obs, y=thr.ets.irt)) +
    geom_point(shape=1, colour="grey50") +  # Use open circles
    geom_smooth(method="lm", se=FALSE,
    formula = y ~ poly(x, 3),
    size = 1) +      # Defines regression line
    dev.new(width=5, height=4) +
    scale_x_continuous(limits=c(0.07, 0.93), breaks=c(0.2,0.4,0.6,0.8)) +
    scale_y_continuous(limits=c(4, 26), breaks=c(5,10,15,20,25))


##### RESIDUALS ANALYSIS

true.ets.tru <- 15.139   # True test score threshold for theta = 0

# Create sub-files

res <- df[df$rel.state.par==0.5 & df$prev.obs<0.3,]
res <- df[df$rel.state.par==0.7 & df$prev.obs<0.3,]
```

```
res <- df[df$rel.state.par==0.9 & df$prev.obs<0.3,]

res <- df[df$rel.state.par==0.5 & df$prev.obs>=0.3 & df$prev.obs<0.5,]
res <- df[df$rel.state.par==0.7 & df$prev.obs>=0.3 & df$prev.obs<0.5,]
res <- df[df$rel.state.par==0.9 & df$prev.obs>=0.3 & df$prev.obs<0.5,]

res <- df[df$rel.state.par==0.5 & df$prev.obs>=0.5 & df$prev.obs<0.7,]
res <- df[df$rel.state.par==0.7 & df$prev.obs>=0.5 & df$prev.obs<0.7,]
res <- df[df$rel.state.par==0.9 & df$prev.obs>=0.5 & df$prev.obs<0.7,]

res <- df[df$rel.state.par==0.5 & df$prev.obs>=0.7,]
res <- df[df$rel.state.par==0.7 & df$prev.obs>=0.7,]
res <- df[df$rel.state.par==0.9 & df$prev.obs>=0.7,]

dim(res)

# Means and 95CI

# ROC-Y
round( mean(res$thr.rocy), 2)
hlp <- res$thr.rocy - true.ets.tru
round( bias <- mean(hlp), 2 )                    # bias
# round( quantile(hlp, prob=c(0.025, 0.975)), 2)
round( MSR <- bias^2 + var(hlp),2 )              # MSR

# Pred
round( mean(res$thr.pred), 2)
hlp <- res$thr.pred - true.ets.tru
round( bias <- mean(hlp), 2 )                    # bias
# round( quantile(hlp, prob=c(0.025, 0.975)), 2)
round( MSR <- bias^2 + var(hlp),2 )              # MSR

# Adjusted 2
round( mean(res$thr.adj,na.rm=T), 2)
hlp <- res$thr.adj - true.ets.tru
round( bias <- mean(hlp,na.rm=T), 2 )            # bias
# round( quantile(hlp, prob=c(0.025, 0.975)), 2)
round( MSR <- bias^2 + var(hlp,na.rm=T),2 )      # MSR

# PVs
round( mean(res$thr.ets.pls), 2)
hlp <- res$thr.ets.pls - true.ets.tru
round( bias <- mean(hlp), 2 )                    # bias
# round( quantile(hlp, prob=c(0.025, 0.975)), 2)
round( MSR <- bias^2 + var(hlp),2 )              # MSR
```

```
# IRT
round( mean(res$thr.ets.irt), 2)
hlp <- res$thr.ets.irt - true.ets.tru
round( bias <- mean(hlp), 2 )                          # bias
# round( quantile(hlp, prob=c(0.025, 0.975)), 2)
round( MSR <- bias^2 + var(hlp),2 )                    # MSR


##### TABLE 1 -- DESCRIPTIVES

data.frame(names(df))

round(mean(df$mn.sum),1)
round(min(df$mn.sum),1)
round(max(df$mn.sum),1)

round(mean(df$sd.sum),1)
round(min(df$sd.sum),1)
round(max(df$sd.sum),1)

round(mean(df$skew.sum),2)
round(min(df$skew.sum),2)
round(max(df$skew.sum),2)

round(mean(df$kurt.sum),2)
round(min(df$kurt.sum),2)
round(max(df$kurt.sum),2)

round(mean(df$flor),2)
round(min(df$flor),2)
round(max(df$flor),2)

round(mean(df$ceil),2)
round(min(df$ceil),2)
round(max(df$ceil),2)

round(mean(df$prev.obs),2)
round(min(df$prev.obs),2)
round(max(df$prev.obs),2)
```

3. Calculation of the true threshold in terms of the expected test score

In the article, we simulated datasets of a hypothetical questionnaire with 10 items with 4 response options (0, 1, 2, and 3) using the IRT-parameters given in the Appendix, and we defined the true threshold of interest in terms of the latent trait as theta $\theta = 0$. Here we calculate the corresponding expected test score of this threshold, based on the IRT-parameters.

The probability of item $\mathrm{X_j}$ scoring ≥ *k* (*k* is 1, 2 or 3) is given by:

$$\ln\left(\frac{P(\mathrm{X_j} \geq k)}{(1 - P(\mathrm{X_j} \geq k))}\right) = a_{\mathrm{j}}(\theta - b_{\mathrm{jk}})$$

where $\ln$ represents the natural logarithm, *P* represents the probability, a_{j} represents the item discrimination parameter and b_{jk} represents the kth difficulty parameter of item j.

The equation can be rewritten as:

$$P(\mathrm{X_j} \geq k) = \frac{e^{a_{\mathrm{j}}(\theta - b_{\mathrm{jk}})}}{1 + e^{a_{\mathrm{j}}(\theta - b_{\mathrm{jk}})}}$$

where e represents the natural exponential function.

The probability of item $\mathrm{X_j}$ scoring = *k* is given by:

$$P(\mathrm{X_j} = k) = P(\mathrm{X_j} \geq k) - P(\mathrm{X_j} \geq k + 1)$$

This way, the probabilities of item $\mathrm{X_j}$ scoring 1 ($P(\mathrm{X_j} = 1)$), 2 ($P(\mathrm{X_j} = 2)$), or 3 ($P(\mathrm{X_j} = 3)$) can be calculated. The expected score of item $\mathrm{X_j}$ then is:

$$P(\mathrm{X_j} = 1) * 1 + P(\mathrm{X_j} = 2) * 2 + P(\mathrm{X_j} = 3) * 3$$

After calculating the expected item scores, the expected test (or scale) score, can be calculated by summing the expected item scores of the scale.

4. R-code for estimating a meaningful threshold using IRT

```
# INSTRUCTIONS
# This code let you estimate an IRT based meaningful threshold in your data.
# Part 1 provides the code for simulating a dataset for illustration.
# Part 2 provides the actual code for estimation.
# If you want to analyze your own data, go to part 2.

library(mirt)

##### PART 1: SIMULATE DATASET

# Create set of item parameters

N <- 1000                 # simulated sample size
nitems <- 10              # number of items
ncat <- 4                 # number of response categories per item

a  <- c(0.6,0.6,0.8,0.8,1,1,1.2,1.2,1.4,1.4)
b2 <- (a-1)*2
bc <- (a-1)/2

set.seed(12345)

a1 <- sample(a)
b1 <- b2 - 1 + sample(bc)
b3 <- b2 + 1 + sample(bc)

cf.simb <- data.frame(a1,b1,b2,b3)
round(cf.simb, 3)
round(colMeans(cf.simb), 3)

# Transform b-parameters to d-parameters
# difficulty (b) = easiness (d) / -a

cf.sim1 <- cf.simb

colnames(cf.sim1) <- c("a1","d1","d2","d3")

cf.sim1$d1 <- -cf.simb$b1*a1
cf.sim1$d2 <- -cf.simb$b2*a1
cf.sim1$d3 <- -cf.simb$b3*a1
round(cf.sim1, 3)              # dysplays the item parameters
round(colMeans(cf.sim1), 3)
```

```
# Simulate dataset using 'mirt'

a1 <- as.matrix(cf.sim1[ , 1])
d1 <- as.matrix(cf.sim1[ , -1])
theta.sim <- as.matrix( rnorm(N, 0, 1) )

dat <- simdata(a1, d1, N, itemtype="graded", Theta=theta.sim)
dat <- as.data.frame(dat)

head(dat)


# Create dichotomous state (i.e., anchor) variable

( rel.st <- 0.5 )     # reliability of the 'perceived trait'
sd.error <- sqrt(((1-rel.st)/rel.st)*sd(theta.sim)^2)
theta.err <- theta.sim + rnorm(N, 0, sd.error)

# State variable and observed prevalence
state <- numeric(N)
state[theta.err > 0] <- 1       # Threshold at theta.sim = 0
( p <- mean(state) )            # State prevalence


# Create dataset with state variable

dat <- data.frame(dat, state)
head(dat)

org <- dat

#########################################################

##### PART 2: ESTIMATION

###  Read your own data

# The following code opens a windows dialogue box in which you can browse
# to you data file.
# Note that your datafile should contain the <nitems> items of your PROM
# and in the last column the dichotomous state (or anchor) variable.

org <- read.table(file.choose(), header=T)  # The file is named "org"

nitems = 10     # Provide the number of items in the scale
```

```
# The file is next copied to a file called "dat" which is the working file
dat <- org

# Fit a graded response model
mod <- mirt(data=dat, model=1, itemtype="graded", TOL=.001)

# Recover the item parameters from the model
( cf <- coef(mod, simplify=TRUE, IRTpars=TRUE)$items )

# Extract the b-parameter of the state variable
( thr.thet <- as.matrix( cf[nitems+1, 2] ) )

# And calculate the corresponding expected test score
expected.test(mod, thr.thet, which.items = 1:nitems)

rm(mod)


##### Empirical bootstrap to find the 95% CI

nboot    <- 1000                # number of bootstrap samples
threshold <- as.numeric(rep(NA, nboot))

start.time <- Sys.time()

for(i in 1:nboot) {

  print(i)

  dat <- org[sample(1:dim(dat)[1], dim(dat)[1], replace=TRUE),]

  cap <- capture.output( mod <- mirt(data=dat, model=1,
                                itemtype="graded", TOL=.001) )

  cf <- coef(mod, simplify=TRUE, IRTpars=TRUE)$items

  ( thr.thet  <- as.matrix( cf[nitems+1, 2] ) )
  ( threshold[i]  <- expected.test(mod, thr.thet, which.items = 1:nitems) )

  rm(mod)
}

end.time <- Sys.time()
time.taken <- end.time - start.time
time.taken
```

```
# NOTE: If you click on the R Console you can see how much bootstrap samples
# have been processed.

### Bootstrap results

round(mean(threshold),1)
round(quantile(threshold, c(0.025, 0.975)),1)

# Show histogram
hist(threshold)
```
